# A Treatise on Diseases of the Eye, Nose, Throat, and Ear

**Published:** 1903-02

**Authors:** 


A Treatise on Diseases of the Eye, Nose, Throat, and Ear. For Students and Practitioners. By various authors. Edited by William Campbell Posey, A. B., M. D., Professor of Ophthalmology in the Philadelphia Polyclinic; Surgeon to the Wills Eye Hospital; Ophthalmic Surgeon to the Howard and Epileptic Hospitals. And Jonathan Wright, M. D., Attending Laryngologist to Kings County Hospital, Brooklyn. Royal 8vo, pp. 1252. Illustrated with 650 engravings and 35 plates in colors and monochrome. Philadelphia and New York: Lea Brothers & Co. 1902. [Cloth, $7.00; leather, $8 00.]

In the preface of this book the editors state that it is especially designed for the general practitioner and the student. That being true, it may be regretted that it is not a better book.

One of the first things to strike the reader's eye, is a typographical error on page 48, where a cut of the physiological excavation of the optic nerve is called the "physiological examination," and one of the last things is an error in spelling in the index where Pray's astigmatic letters are said to be Progs. Such faults are particularly deplorable in a work which has for its prime object, the instruction of the uninitiated; both on account of their being misleading and because when discovered, they are apt to breed distrust of the whole text.

Excepting those of the fundus oculi, the plates are all poorly colored and, consequently not as good as the other illustrations which are less pretentious. The eye, nose and throat have a fair share of space devoted to them; but the ear is positively slighted.

Drs. Weeks, Duane and Wood have contributed excellent articles and to say that they are fully up to the standards of these well known writers, is the only comment necessary.

The crowning feature of the whole volume is The Eye in Its Relations to General Diseases, written by Dr. Clark. Besides being both instructive and attractive, it is a very convenient and valuable source of reference, that will be greatly appreciated by busy men who heretofore have been obliged to search through a number of different chapters in order to find what this writer has condensed into one. It is a most commendable idea.

As a whole, the book is not pleasing; it is far from perfect and gives one the impression that it was published hurriedly. It will bear a deal of pruning and should be much better in its second edition. L. B.

